# Epitranscriptomic advances in normal and malignant hematopoiesis

**DOI:** 10.1038/s41375-025-02765-6

**Published:** 2025-09-19

**Authors:** Maria Eleftheriou, James Russell, Konstantinos Tzelepis

**Affiliations:** 1 Cambridge Stem Cell Institute, University of Cambridge, Cambridge, UK; 2 Department of Haematology, University of Cambridge, Cambridge, UK; 3 Milner Therapeutics Institute, University of Cambridge, Cambridge, UK; 4 Wellcome Trust Sanger Institute, Cambridge, UK

**Keywords:** Haematological cancer, Cancer stem cells, Translational research, Oncogenesis

## Abstract

RNA modifications, collectively termed the epitranscriptome, constitute a dynamic layer of post-transcriptional regulation that governs RNA splicing, stability, localization, translation, and decay. In the hematopoietic system, these chemical marks influence stem cell fate, lineage specification, immune surveillance, and malignant transformation through context-dependent regulation of mRNA, tRNA, rRNA, and non-coding RNAs. Here, we focus on RNA modifications and editing events with emerging mechanistic and translational relevance in normal and malignant hematopoiesis, highlighting those implicated in stem cell dynamics, leukemic progression, and therapeutic resistance. Specifically, we discuss N⁶-methyladenosine (m⁶A), 5-methylcytosine (m⁵C), N⁷-methylguanosine (m⁷G), N⁴-acetylcytidine (ac⁴C), pseudouridine (Ψ), adenosine-to-inosine (A-to-I) editing, and RNA glycosylation. Particular attention is given to enzymes such as METTL3, METTL1, ADAR1, and NAT10, whose dysregulation sustains leukemic stem cell programmes, promotes immune evasion, and confers treatment resistance. With the first-in-class METTL3 inhibitor STC-15 now in early-phase clinical trials in solid tumours (NCT05584111, NCT06975293), and additional RNA-modifying enzyme inhibitors advancing preclinically, these pathways are emerging as therapeutically tractable, including in hematological cancers. Furthermore, integrating epitranscriptomic profiles into genomic risk frameworks may also improve disease stratification, minimal residual disease (MRD) monitoring, and the identification of targetable vulnerabilities. Together, these insights position RNA modifications as central to blood cancer biology and support their integration into next-generation diagnostic, prognostic, and therapeutic strategies.

## Introduction

Chemical modifications of DNA, RNA, and proteins are fundamental regulators of gene expression during mammalian development and disease. Although RNA modifications were first described in the 1950s [1], their functional relevance has only recently come into focus. Collectively termed the epitranscriptome, these modifications constitute a dynamic regulatory layer that influences RNA fate through control of splicing, stability, translation, and localization [2]. Among the most studied RNA modifications, N⁶-methyladenosine (m⁶A), 5-methylcytosine (m⁵C), pseudouridine (Ψ), N⁷-methylguanosine (m⁷G), and N⁴-acetylcytidine (ac⁴C) play prominent roles in post-transcriptional gene regulation [2]. RNA editing, particularly adenosine-to-inosine (A-to-I) conversion, further contributes to transcript diversity and adaptive cellular responses.

Epitranscriptomic modifications are critical regulators of hematopoietic stem cell fate, modulating self-renewal, differentiation, and lineage specification. Among these, m⁶A is the most abundant and well-characterized internal mRNA modification, and the most extensively studied in hematopoiesis. It is dynamically installed, interpreted, and removed by well-orchestrated writer, reader, and eraser proteins, which together regulate the function of hematopoietic stem and progenitor cells (HSPCs) [3, 4]. In malignant contexts, dysregulation of such epitranscriptomic pathways perturbs gene expression programs, promoting oncogenic transformation, immune evasion, and leukemic progression [5].

Recent advances in next-generation sequencing and multi-omics profiling have deepened our understanding of the epitranscriptome in hematological malignancies. Transcriptome-wide mapping at single-nucleotide resolution has uncovered context-specific modification landscapes in both normal HSPCs and leukemia cells [6]. Functional studies further demonstrate that m⁶A regulators are essential for maintaining leukemic self-renewal, fueling interest in the therapeutic targeting of RNA-modifying enzymes, with first-in-class small-molecule inhibitors now advancing through hematology pipelines.

**Fig. 1:**
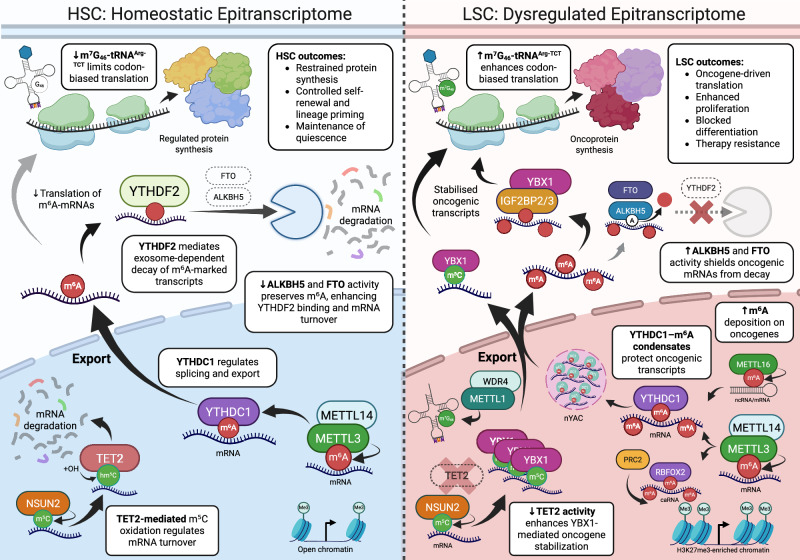
Divergent regulation of the epitranscriptome in HSCs and LSCs. In hematopoietic stem cells (HSCs, left), METTL3–METTL14 deposits N⁶-methyladenosine (m⁶A), supporting open chromatin and balanced transcript methylation. Nuclear YTHDC1 promotes accurate splicing and regulated export, while cytoplasmic YTHDF2 mediates degradation of m⁶A-marked transcripts. Low expression of IGF2BP2/3 and YBX1, together with reduced ALKBH5 and FTO activity, preserves m⁶A marks and facilitates YTHDF2-directed decay. Nuclear NSUN2-mediated m⁵C deposition and TET2-dependent oxidation promote mRNA turnover. Protein synthesis is restrained, supporting regulated self-renewal, lineage priming, and quiescence. In leukemic stem cells (LSCs, right), METTL3 is upregulated, enhancing m⁶A deposition on oncogenic transcripts. Nuclear YTHDC1 forms condensates (nYACs) that shield m⁶A-modified RNAs from decay. METTL16 is also upregulated, contributing to m⁶A modification of structured coding and non-coding RNAs. RBFOX2 binds m⁶A-marked chromatin-associated RNAs (caRNAs), recruiting PRC2 and promoting focal H3K27me3 deposition. TET2 loss increases m⁵C levels, stabilized by nuclear YBX1. In the cytoplasm, IGF2BP2/3 and YBX1 reinforce stabilization of oncogenic mRNAs, while elevated ALKBH5 and FTO activity removes m⁶A, limiting YTHDF2-mediated decay. Nuclear METTL1–WDR4 upregulation increases m⁷G modification of Arg-TCT tRNAs, enhancing codon-biased translation of AGA-rich oncogenes. These coordinated changes promote oncoprotein synthesis, sustaining LSC self-renewal, immune evasion, and therapy resistance. m⁶A N⁶-methyladenosine, m⁵C 5-methylcytosine, HSC hematopoietic stem cell, LSC leukemic stem cell, YTHDC1 YTH domain-containing protein 1, YTHDF2 YTH domain family protein 2, IGF2BP insulin-like growth factor 2 mRNA-binding protein, YBX1 Y-box binding protein 1, ALKBH5 AlkB homolog 5, FTO fat mass and obesity-associated protein, NSUN2 NOP2/Sun RNA methyltransferase 2, TET2 ten-eleven translocation methylcytosine dioxygenase 2, METTL1 methyltransferase-like protein 1, WDR4 WD repeat domain 4, METTL3 methyltransferase-like protein 3, METTL14 methyltransferase-like protein 14, METTL16 methyltransferase-like protein 16, RBFOX2 RNA binding fox-1 homolog 2, PRC2 polycomb repressive complex 2, caRNA chromatin-associated RNA, nYAC nuclear YTHDC1–m⁶A condensate, tRNA transfer RNA. Created with BioRender.com.

In this review, we summarise emerging insights into the role of RNA modifications in normal and malignant hematopoiesis, with emphasis on mechanistic pathways and potential therapeutic opportunities (Fig. 1).

## The role of m⁶A in normal and malignant hematopoiesis

N⁶-methyladenosine (m⁶A) is one of the most abundant RNA modifications in mammals. It is added at the nitrogen-6 position of adenosine residues by the N⁶-adenosine-methyltransferase complex (METTL3–METTL14), on several RNA fractions, but mainly on mRNA. The m⁶A mark regulates transcript stability, splicing, and translation [7], and plays essential roles in both normal hematopoiesis and leukemogenesis [8]. Epitranscriptomic profiling across hematopoietic stages reveals m⁶A enrichment during early development, while it stabilizes transcripts required for hematopoietic stem cell (HSC) maintenance [9]. These findings position m⁶A as a key regulator of the HSC transcriptional state, particularly in response to environmental stimuli [9].

Multiple studies support a role for m⁶A in balancing HSC self-renewal and differentiation [3]. One study identified *MYC* as an m⁶A-modified transcript, with methylation enhancing its stability and translation in HSCs, thereby sustaining MYC-driven programs that promote self-renewal and restrain premature differentiation [3]. Another study identified *SON* as an m⁶A-modified transcript that supports HSC self-renewal and symmetric commitment under inflammatory conditions [10]. Loss of m⁶A destabilizes *SON* mRNA, leading to upregulated inflammatory signaling, premature differentiation, and disruption of asymmetric division [10].

The m⁶A RNA modification is also critical during the endothelial-to-hematopoietic transition (EHT) [11]. In *Mettl3*-deficient embryos, global m⁶A levels are reduced, impairing the development and differentiation of HSPCs [11]. Conditional deletion of *Mettl3* in the foetal liver results in hematopoietic failure and perinatal lethality [12]. Mechanistically, *Mettl3* loss activates an aberrant innate immune response, triggered by the accumulation of endogenous double-stranded RNAs (dsRNA), typically associated with viral mimicry [12, 13]. These findings suggest that m⁶A suppresses inappropriate dsRNA accumulation and preserves immune quiescence during hematopoiesis [12].

## Regulators of m⁶A: writers, erasers, and translational control

The m⁶A methyltransferase complex METTL3–METTL14 functions as a heterodimer to install m⁶A on poly-A-enriched RNA, with METTL3 serving as the catalytically active subunit [14]. The enzymes ALKBH5 and FTO remove m⁶A through Fe(II)/α-ketoglutarate-dependent dioxygenase activity [8]. The demethylase ALKBH5 directly catalyzes the removal of m⁶A, converting it to adenosine, while the hydroxylase FTO sequentially oxidizes m⁶A to hydroxymethyladenosine (hm⁶A) and formyladenosine (f⁶A), illustrating divergent enzymatic pathways and potentially distinct cellular functions. METTL3 contributes to translational regulation through both methylation-dependent and -independent mechanisms; notably, it can enhance translation of specific target mRNAs independently of m⁶A [15].

Core components of the m⁶A machinery, including METTL3, METTL14, FTO, and ALKBH5, have been implicated in acute myeloid leukemia (AML) pathogenesis [8, 16–19]. AML is characterized by clonal expansion of HSCs and HSPCs, impaired myeloid differentiation, and the emergence of self-renewing leukemic stem cells (LSCs) [20, 21]. Knockdown of METTL14 promotes myeloid differentiation of normal HSPCs and impairs AML cell growth and self-renewal in vitro and in vivo [22]. These effects are partly mediated by the destabilization of key m⁶A-modified transcripts such as *MYB* and *MYC* [22]. METTL14 knockdown may secondarily reduce METTL3 expression, disrupting the stability and function of the METTL3–METTL14 writer complex.

METTL3 has been identified as an essential gene for AML cell survival in genome-wide CRISPR-Cas9 screens [16, 23]. Promoter-bound METTL3 catalyzes m⁶A deposition within coding regions of oncogenic transcripts, enhancing their translation [16]. These targets are critical for leukemia maintenance, positioning METTL3 as a compelling therapeutic target in AML [16]. Additionally, METTL3 is upregulated in chemotherapy-resistant AML cells, where it contributes to resistance by promoting bone marrow homing and engraftment through upregulation of *ITGA4* [24]. In myelodysplastic neoplasms (MDS) with *DDX41* mutations, the nuclear m⁶A reader YTHDC1 interacts with the METTL3–METTL14 complex to maintain genomic stability [25]. RNA-related stability is caused by R-loops, and recent studies have shown the presence of m⁶A modifications on R-loops and their role in maintaining stability [26]. In the context of *DDX41* mutation, YTHDC1 binding to METTL3–METTL14 is impaired, leading to R-loop accumulation, genomic instability, and disease progression [25].

Beyond METTL3 and METTL14, METTL16 is an m⁶A methyltransferase that primarily modifies non-coding RNAs (ncRNAs). METTL16 has also been identified as essential for AML cell survival in genome-wide CRISPR-Cas9 screens [27]. In vitro and in vivo studies show that METTL16 knockdown impairs AML initiation and progression, and restricts leukemic infiltration of the bone marrow, spleen, liver, and peripheral blood [27]. Mechanistically, METTL16 methylates mRNAs encoding branched-chain amino acid (BCAA) metabolic enzymes *BCAT1* and *BCAT2* in human AML cells, but not in normal HSPCs. This promotes BCAA catabolism, which is essential for AML cell survival [27]. YTHDC1 binds to m⁶A-marked *BCAT1* and *BCAT2* transcripts, enhancing their stability and expression. These findings suggest a cooperative mechanism in which METTL16 installs m⁶A and YTHDC1 acts to maintain metabolic transcript output [27].

The erasers FTO and ALKBH5 are frequently upregulated in AML and promote leukemogenesis by demethylating oncogenic transcripts [18, 28]. ALKBH5 is overexpressed in primary AML patient samples, and its knockdown or inhibition increases global m⁶A levels, suppresses proliferation, and induces apoptosis [14, 28, 29]. ALKBH5 demethylates *AXL* mRNA, stabilizing it and enhancing translation, thereby supporting LSC survival [29]. FTO is highly expressed in AML cases with KMT2A (formerly *MLL*) rearrangements and may act as a downstream target of KMT2A fusion proteins [18]. Also, FTO overexpression leads to oncogenic transformation both in vitro and in vivo, and suppresses all-trans retinoic acid (ATRA)-induced differentiation by reducing m⁶A on *ASB2* and *RARA* transcripts, resulting in their downregulation and diminished ATRA responsiveness [18].

## Regulators of m⁶A: readers

YTHDC1 is a nuclear m⁶A reader that regulates mRNA splicing and nuclear export [30]. A recent genome-wide CRISPR-Cas9 screen identified YTHDC1 as the highest-ranked m⁶A reader in AML models [31]. Functional studies confirm that YTHDC1 is required for leukemia maintenance and LSC self-renewal [32]. RNA-sequencing of *YTHDC1*-depleted AML cells revealed downregulation of several transcripts, including *MCM4* (Mini-Chromosome Maintenance Complex 4), a core DNA replication factor [32]. YTHDC1 stabilizes *MCM4* mRNA in an m⁶A-dependent manner, and *MCM4* depletion leads to DNA damage and genomic instability while forced expression of *MCM4* in YTHDC1-deficient cells restores proliferation [32]. Beyond transcript stabilization, m⁶A is required for YTHDC1 to undergo liquid-liquid phase separation and form nuclear YTHDC1–m⁶A condensates (nYACs) [31]. These condensates are more abundant in AML cells than in normal HSC and HSPC cohorts, and help sustain leukemic cell survival by shielding m⁶A-modified transcripts from degradation by the PAXT complex and the RNA exosome [31]. Importantly, *YTHDC1* knockdown (KD) in primary AML cells caused growth inhibition without affecting the growth of healthy CD34^+^ cells [32].

YTHDF1 and YTHDF3 are cytoplasmic m⁶A readers that promote translation of target mRNAs [33]. In contrast, YTHDF2 facilitates degradation of m⁶A-modified transcripts [34]. YTHDF2 is highly expressed across diverse AML subtypes and contributes to LSC maintenance by modulating turnover of m⁶A-marked transcripts [35]. It is particularly enriched in CD34^+^ AML fractions, and conditional knockout impairs colony formation upon serial replating [35]. YTHDF2 knockdown also reduces proliferation and induces apoptosis in AML models, without affecting myeloid differentiation [35]. YTHDF2 is also highly expressed in multiple myeloma (MM), where it promotes proliferation through m⁶A-dependent degradation of *EGR1* mRNA, thereby disrupting the EGR1/p21^cip1/waf1^/CDK2–Cyclin E1 axis. It is also an independent prognostic marker in MM, with high expression associated with advanced stage, relapse, treatment resistance, and poor overall survival [36]. *Ythdf2* deletion in mice does not significantly disrupt steady-state hematopoiesis, with HSPC populations largely preserved [35]. The long-term effects of YTHDF2 loss were assessed in secondary transplantation assays using CD45.2⁺Lin⁻Sca-1⁺c-Kit⁺ (LSK) cells from primary mouse recipients; YTHDF2-deficient cells failed to maintain multilineage hematopoiesis and exhibited a myeloid bias [37]. Transcriptomic analysis of *YTHDF2*-deficient cells revealed upregulation of inflammatory gene programs, including type I and type II interferon responses, alongside broader pro-inflammatory signaling [37]. These findings suggest that YTHDF2-mediated, m⁶A-dependent RNA degradation acts as a protective mechanism by limiting inflammatory signaling and preserving normal HSC function [37]. Recent studies have shown considerable functional overlap between the m⁶A readers YTHDF1-3, suggesting both redundancy and specialized roles depending on the cellular context. However, the significance of these findings remains an area of ongoing investigation in both normal and disease states [38–40].

The insulin-like growth factor-binding proteins (IGF2BPs) are cytoplasmic m⁶A readers that enhance mRNA stability and translation. IGF2BP3 is overexpressed in AML and correlates with adverse clinical outcomes [41, 42]. IGF2BP3 loss suppresses proliferation, induces apoptosis, and impairs the leukemogenic potential of AML cells in vitro and in vivo by stabilizing m⁶A-modified *RCC2* mRNA, which encodes a key regulator of cell cycle progression [42]. IGF2BP2 also contributes to AML maintenance and has been identified as a regulator of HSC function by binding m⁶A-modified transcripts and promoting their stability and translation [9]. *IGF2BP2* knockdown reduces colony-forming potential of AML progenitor cells, and *Igf2bp2* loss delays disease progression in secondary transplant models, consistent with a functional role in LSC maintenance [41]. Protein arginine methyltransferase 6 (PRMT6) has been proposed as a downstream effector of IGF2BP2. Loss of PRMT6 selectively impairs LSC function without disrupting normal hematopoiesis [41]. PRMT family enzymes catalyze arginine methylation of histones and other proteins, and several members have been implicated in hematological malignancies [43, 44]. Pharmacological inhibition of PRMT6 using EPZ020411 suppresses AML progression and impairs LSC function via an m⁶A-dependent mechanism [41]. IGF2BPs have also been shown to cooperate with the RNA-binding protein YBX1 to mediate recognition of m⁶A-modified transcripts. Knockdown of IGF2BP1 or IGF2BP3 disrupts YBX1 binding to m⁶A-tagged mRNAs, including *MYC* and *BCL2*, leading to transcript destabilization and impaired AML cell survival [45].

RBFOX2 was recently identified as a nuclear m⁶A reader that binds chromatin-associated RNAs (caRNAs) in AML [46, 47]. It recognizes m⁶A-modified caRNAs and recruits the adapter protein RBM15, which facilitates further m⁶A deposition on promoter-associated RNAs [46, 47]. These RNAs subsequently engage YTHDC1, enabling recruitment of Polycomb Repressive Complex 2 (PRC2) to mediate transcriptional silencing and support LSC maintenance [46, 47]. Collectively, these findings position RBFOX2 as a functionally distinct m⁶A reader with emerging therapeutic relevance in AML.

## Therapeutic targeting of m⁶A RNA modifiers in AML

Over the past decade, genes involved in m⁶A regulation have emerged as promising therapeutic targets in hematological and solid malignancies. Recent studies explore the development of small-molecules that block the catalytic activity of METTL3 [48]. However, a major therapeutic advance in the field of epitranscriptomics was the development of the first-in-class RNA methyltransferase inhibitor STM2457, a potent, selective, and bioavailable METTL3 inhibitor [17]. STM2457 targets the METTL3–METTL14 complex and inhibits the catalytic activity of METTL3, evident by the reduced global m⁶A levels following treatment [17]. In AML models, STM2457 impaired leukemic cell growth and promoted differentiation and apoptosis in vitro and in vivo [17]. Mechanistically, it reduced m⁶A deposition on key leukemogenic transcripts, thereby impairing their translation without affecting transcript abundance [17]. These findings provided the first proof-of-concept for pharmacological inhibition of RNA-modifying enzymes as a viable therapeutic strategy in AML. Follow-up studies revealed that METTL3 inhibition also impacts normal hematopoiesis [49]. Treatment with STM2457 destabilized lineage-determining transcripts in hematopoietic stem cells (HSCs), impairing erythroid differentiation and highlighting anemia as a potential on-target effect [49]. A successor compound, STC15, is currently under clinical evaluation in Phase 1a (NCT05584111) and Phase 1b/2 (NCT06975293) trials, which has shown early signs of tolerability and anti-cancer efficacy [50].

Another translational advance was the development of two small-molecule FTO inhibitors, FB23 and FB23-2, which blocked its demethylase activity and suppressed AML cell growth while promoting differentiation and apoptosis in vitro and in vivo [51]. Subsequent experiments using these small molecules demonstrated that FTO inhibition can enhance the efficacy of conventional chemotherapy in AML models [51]. However, these small molecules exhibited low specificity and selectivity. More recently, two additional FTO inhibitors, CS1 and CS2, were developed with increased potency and anti-leukemic activity [19]. In this study, both pharmacological and genetic inhibition of FTO reduced LSC self-renewal and downregulated immune checkpoint expression in AML samples. FTO inhibition also sensitized AML cells to T-cell-mediated cytotoxicity and reversed immune evasion induced by hypomethylating agents [19]. Despite the encouraging anti-leukemic activity in preclinical models, the therapeutic impact of FTO inhibition remains to be investigated in clinical trials.

## 5-methylcytosine (m⁵C) RNA modification

5-methylcytosine (m⁵C) is a widespread epitranscriptomic mark that regulates RNA metabolism, including transcript stability, translation, and nuclear export. Its deposition is catalyzed by the NSUN family of RNA methyltransferases, comprising seven members (NSUN1–NSUN7). Among these, NSUN2 acts primarily on tRNAs, installing m⁵C at the anticodon and variable loops to protect against endonucleolytic cleavage into tRNA-derived fragments (tRFs), thereby sustaining global translation under cellular stress [52, 53]. NSUN2 also methylates mRNAs, where m⁵C stabilizes transcripts encoding key metabolic enzymes, including PHGDH and SHMT2, which are essential for serine/glycine biosynthesis and are implicated in acute myeloid leukemia (AML) progression and leukemic stem cell (LSC) self-renewal [54]. NSUN5, by contrast, modifies rRNAs to regulate ribosome function and translational selectivity [55]. m⁵C is removed from mRNAs by Ten-Eleven Translocation (TET) family dioxygenases through stepwise oxidative demethylation⁴⁸.

Beyond NSUN2, disruption of other m⁵C-associated proteins, including writers, erasers, and putative readers, has also been implicated in hematological malignancies. In B-cell lymphoma, a prognostic model incorporating m⁵C signatures across selected mRNAs delineated molecular subtypes with distinct m⁵C landscapes, tumor microenvironmental features, and clinical outcomes [56].

In AML and MDS, the RNA methyltransferases NSUN3 and DNMT2 sensitize malignant cells to 5-azacytidine (5-Aza), a hypomethylating agent widely used in clinical practice [57]. Mechanistically, these enzymes interact with hnRNPK, an RNA-binding protein that recruits transcription factors to remodel chromatin and enhance 5-Aza accessibility [57]. In contrast, NSUN1 promotes resistance to 5-Aza by supporting BRD4 and RNA polymerase II recruitment to form a transcriptionally active, drug-refractory chromatin state [57]. These findings suggest that targeting specific m⁵C methyltransferases could enhance therapeutic responses to hypomethylating agents.

*SRSF2* mutations are common in AML, MDS, and chronic myelomonocytic leukemia (CMML) [58]. The most frequent variant is a proline-to-histidine substitution at codon 95 (P95H) [59]. In addition to its role in RNA splicing, SRSF2 also functions as a reader of m⁵C-modified transcripts. The P95H mutation impairs this binding, particularly to transcripts involved in leukemogenic pathways, leading to altered splicing patterns [60]. A strong association has been observed between the SRSF2 P95H mutation, reduced NSUN2 expression, and adverse prognosis in AML [60]. In NSUN2-deficient K562 cells, global m⁵C levels decline, and mutant SRSF2 exhibits reduced binding to m⁵C-marked transcripts [60]. These findings highlight the complex functional and clinical significance of an interaction between the disrupted m⁵C and a splicing factor in AML pathogenesis.

TET2 is frequently mutated or downregulated in AML, where it plays key roles in both hematopoiesis and leukemogenesis [61]. In addition to its canonical role in DNA demethylation, TET2 also acts as an RNA demethylase, oxidatively removing m⁵C marks [61]. Loss of TET2 increases m⁵C levels on mRNA and correlates with inferior survival in hematological malignancies [62, 63]. In HSPCs, TET2 deficiency induces chromatin decompaction and genomic instability, promoting myeloid transformation [64]. Transcriptomic profiling of *Tet2*-deficient AML mouse models revealed upregulation of genes associated with hematopoietic stem cell programs [65]. Colony-forming unit (CFU) assays showed that *Tet2*-deficient pre-LSCs form larger, more proliferative colonies in vitro [65]. In vivo, *Tet2* loss enhances the homing and migration of LSCs to stromal niches, which promotes self-renewal and accelerates leukemogenesis [65]. Mechanistically, the m⁵C-modified transcript *Tspan13* has been identified as a direct downstream target of TET2 [65]. In *TET2*-deficient cells, m⁵C accumulates on *Tspan13* mRNA, which is recognized by the m⁵C-binding protein YBX1, resulting in transcript stabilization and increased expression [65]. Upregulated *Tspan13* activates the CXCR4–CXCL12 axis within the bone marrow microenvironment, enhancing AML cell homing, engraftment, and disease progression [65].

Beyond transcript-level effects, m⁵C marks also occur on chromatin-associated RNAs (caRNAs), where they influence chromatin architecture and RNA metabolism, particularly in glioma [66]. Building on these findings, TET2-mediated m⁵C oxidation of caRNA was examined in AML models, where *TET2* deficiency led to elevated caRNA m⁵C levels, increased global transcription, and enhanced chromatin accessibility, consistent with a role in disease progression [67]. Conversely, *NSUN2* depletion reduced caRNA m⁵C levels and promoted chromatin condensation. These repressive domains overlapped with, and inversely correlated to, the open chromatin regions in TET2-deficient cells, underscoring a regulatory interplay between m⁵C dynamics and chromatin state [67]. At the molecular level, TET2-dependent m⁵C oxidation on caRNAs impairs recruitment of MBD6 and reduces local enrichment of H2AK119ub (ubiquitinated histone H2A at lysine 119), a repressive histone mark [67]. Collectively, these data emphasize the importance of the m⁵C RNA modification not only during normal hematopoiesis but also in disease development and progression.

## N⁷-methylguanosine (m⁷G) RNA modification

N⁷-methylguanosine (m⁷G) is a conserved RNA modification found at the 5′ cap of mRNAs and at internal positions on tRNAs. In mRNA, m⁷G is deposited co-transcriptionally at the 5′ end and promotes RNA stability, nuclear export, and translation [68]. In the tRNA fraction, m⁷G is catalyzed by the METTL1–WDR4 methyltransferase complex, which regulates tRNA structure and decoding fidelity [69].

METTL1–WDR4 expression is often upregulated in AML and correlates with disease progression [70]. Knockdown of *METTL1* reduces global m⁷G levels on tRNAs and impairs protein synthesis, leading to cell cycle arrest and reduced proliferation in AML models [71]. Mechanistically, *METTL1* gain-of-function increases m⁷G levels on specific tRNAs, including the tRNA *Arg-TCT*, which decodes AGA codons. This promotes preferential translation of AGA-enriched transcripts, including those of key oncogenes such as *CDK4*, *HMGA2*, *ASH2L*, *SETDB1*, and *UBE2T*, thereby activating proliferative and self-renewal programs [71]. Consistently, *METTL1* knockdown reduces *BCL2* expression and induces apoptosis via caspase-3 activation, supporting a pro-survival role in AML [70]. Together, these findings suggest that METTL1-dependent m⁷G tRNA methylation remodels the tRNA epitranscriptome to drive codon-biased translation leading to oncogenic transformation and leukemogenesis [70, 71].

Beyond mRNAs and tRNAs, m⁷G RNA methylation has also been implicated in the regulation of long non-coding RNAs (lncRNAs) and circular RNAs (circRNAs) in AML. These non-coding RNA species are increasingly recognized as contributors to disease progression, therapeutic resistance, and clinical outcome [72]. In drug-resistant AML models, transcriptome-wide profiling revealed elevated m⁷G methylation on lncRNAs compared to drug-sensitive controls, accompanied by transcriptional upregulation of resistance-associated genes [73]. Clustering of m⁷G-marked transcripts at AML-relevant loci delineated distinct epitranscriptomic subgroups with potential prognostic value [74]. Similarly, circRNAs have been linked to chemoresistance and disease stratification in AML [75]. In resistant cells, the m⁷G RNA methylation is enriched in exon-derived circRNAs, suggesting a role for modified circRNA in regulating resistance-related gene networks [76].

Together, these findings position m⁷G as an important regulator of non-coding RNA function in AML, with potential applications in prognostication as well as the development of epitranscriptomic biomarkers and therapeutic targets.

## N⁴-acetylcytidine (ac⁴C) RNA acetylation

N-acetyltransferase 10 (NAT10) catalyzes the deposition of N⁴-acetylcytidine (ac⁴C) on tRNA, rRNA, and mRNA [77]. This reaction uses acetyl–CoA as the acetyl donor and is energetically driven by ATP hydrolysis [78, 79]. ac⁴C promotes RNA metabolism by enhancing transcript stability and translational efficiency [78]. NAT10 is significantly overexpressed in bone marrow samples from newly diagnosed AML patients, particularly those with *NPM1* mutations [80]. Elevated NAT10 expression correlates with poor prognosis and resistance to chemotherapy [81]. Genetic or pharmacological inhibition of NAT10 induces cell cycle arrest, suppresses proliferation, and promotes apoptosis in AML models [81].

NAT10 is also upregulated in multiple myeloma (MM), a malignancy characterized by clonal plasma cell expansion in the bone marrow [82]. Its overexpression in MM patient samples is associated with increased cellular proliferation and promotes cell growth in vitro and in vivo [83, 84]. Transcriptomic profiling of *NAT10*-overexpressing MM cells reveals enrichment of proliferation- and cell cycle-associated gene signatures, including an increased G₂/M phase fraction [83, 84]. This phenotype correlates with elevated ac⁴C levels within coding sequence (CDS) regions, suggesting that NAT10-mediated acetylation enhances mRNA stability and translational output [83]. Mechanistically, NAT10 increases ac⁴C deposition on *CEP170* mRNA, enhancing its translation and contributing to malignant progression [83]. It also acetylates *BCL2L1* (BCL-XL) mRNA, activating PI3K–AKT and CDK4/6 signaling to promote MM cell proliferation [84]. Notably, remodelin, a small-molecule NAT10 inhibitor originally studied in solid tumors, demonstrates anti-myeloma activity in preclinical models [83, 84]. Together, these findings position NAT10 and its ac⁴C-associated functions as emerging therapeutic targets in multiple myeloma and other hematological malignancies.

## Pseudouridine (Ψ) RNA modification

Pseudouridine (Ψ) is one of the most abundant and evolutionarily conserved RNA modifications. It is catalyzed by pseudouridine synthases (PUSs), including PUS1, PUS3, PUS7, along with their paralogues PUSL1 and PUSL7, as well as TRUB1 and TRUB2 [85–88]. DKC1, the catalytic subunit of the H/ACA small nucleolar ribonucleoprotein (snoRNP) complex, mediates site-specific pseudouridylation on rRNA and stabilizes the telomerase complex [85, 86]. In chronic lymphocytic leukemia (CLL), reduced *DKC1* expression is associated with defective ribosome biogenesis [85].DKC1 is also required for translational fidelity, particularly during start-site recognition; its inactivation disrupts ribosome function and impairs differentiation in normal HSCs [89]. Recent studies implicate PUS7 in myelodysplastic syndromes (MDS), where chromosome 7 deletions are frequent [87, 88]. PUS7-mediated pseudouridylation regulates the production of tRNA-derived small RNAs (tsRNAs) that influence HSPC fate by modulating translation [78]. Loss of PUS7 disrupts this axis, leading to defective hematopoiesis in both in vitro and in vivo models [87, 88].

## Adenosine-to-Inosine (A-to-I) RNA editing

Adenosine-to-inosine (A-to-I) RNA editing is a post-transcriptional modification in which adenosine residues are deaminated to inosine within double-stranded RNA (dsRNA) structures. This reaction is catalyzed by adenosine deaminases acting on RNA (ADARs) via hydrolytic deamination at the C6 position of adenine [90]. Because inosine is interpreted as guanosine during translation and base pairing, A-to-I editing can alter codon identity, splicing patterns, and RNA secondary structures [90].

Dysregulated A-to-I editing, particularly due to aberrant ADAR1 expression, has been implicated in malignant transformation and altered HSC dynamics [91]. Overexpression of the interferon-inducible ADAR1p150 isoform enhances self-renewal capacity in human cord blood HSPCs [92]. Mechanistically, ADAR1-mediated editing impairs miR-26a maturation, leading to suppression of CDKN1A and acceleration of cell cycle progression in HSPCs [93]. This axis promotes the expansion of malignant progenitor populations. Beyond cell-intrinsic roles, ADAR1 also promotes immune evasion in hematological malignancies. Elevated ADAR1p150 expression induces alternative splicing of STAT3, favoring production of the STAT3β isoform. This variant enhances pre-leukemic stem cell (pre-LSC) survival and facilitates immune escape [94]. In T-cell acute lymphoblastic leukemia (T-ALL), ADAR1 expression correlates with disease recurrence and is essential for the maintenance and survival of leukemia-initiating cells (LICs) in vitro and in vivo [95].

Collectively, these findings position ADAR1 as a central regulator of RNA editing in hematological malignancies, linking A-to-I conversion to progenitor expansion, immune evasion, and therapeutic resistance. These pathways present new opportunities for diagnostic, prognostic, and therapeutic intervention.

## RNA glycosylation: an emerging player in the field

**Fig. 2:**
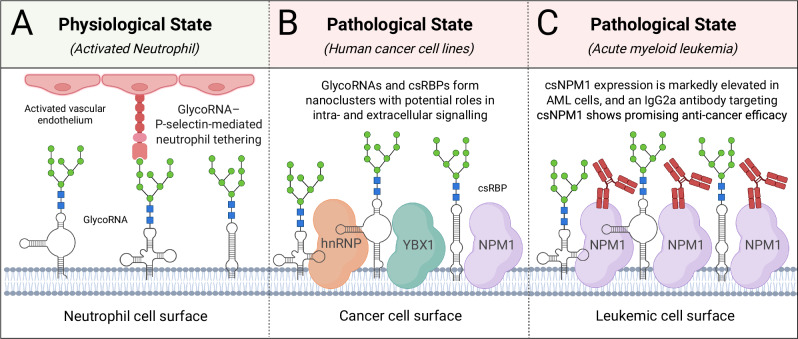
Glycosylated RNAs and cell-surface RNA-binding proteins illustrate emerging roles in immunology and cancer. **A** GlycoRNAs on the surface of neutrophils mediate tethering to activated endothelium via P-selectin. **B** In human cancer cells, glycoRNAs form nanoclusters with cell-surface RNA-binding proteins (csRBPs). **C** On AML cells, glycoRNAs form nanoclusters with cell-surface nucleophosmin 1 (csNPM1). A newly developed IgG2a monoclonal antibody targeting csNPM1 shows anti-leukemic activity in preclinical AML models. AML acute myeloid leukemia, GlycoRNA glycosylated RNA, IgG2a immunoglobulin G2a. Created with BioRender.com.

RNA glycosylation has recently been identified as a novel post-transcriptional modification in mammalian cells (Fig. 2). Small non-coding RNAs can be covalently modified with sialylated N-glycans to generate glycosylated RNAs (glycoRNAs), which localize to the cell surface and participate in immune modulation and extracellular signaling (Fig. 2A) [96]. GlycoRNAs have since been detected on neutrophils, where they regulate recruitment to inflammatory sites. Enzymatic removal of surface RNA impairs neutrophil migration, indicating a functional requirement for glycoRNAs in immune cell trafficking [97].

Subsequently, a class of cell surface RNA-binding proteins (csRBPs) has been identified that form nanoclusters enriched in glycoRNAs (Fig. 2B) [98]. These csRBPs specifically recognize glycosylated RNAs, and their organization is disrupted by RNase treatment, suggesting a structural dependence on intact RNA [98]. This interaction between glycoRNAs and csRBPs facilitates extracellular molecular recognition and intercellular communication, with emerging relevance to immune regulation and therapeutic intervention [98].

Further studies have shown that csRBPs colocalized with glycoRNAs on cancer cells can act as tumor-associated antigens [99]. Among these, full-length nucleophosmin 1 (NPM1) was detected at the cell surface (csNPM1) across multiple tumor models [99]. Although NPM1 is normally confined to the nucleolus, mutant forms commonly observed in AML are aberrantly localized to the cytoplasm, a defining feature of NPM1-mutated leukemias [100]. This is the first report showcasing full-length NPM1 as a cell surface protein, extensively presented on LSCs and various other malignant cells but not on their normal counterparts [99]. Targeting csNPM1 with a monoclonal IgG2a antibody (mAb2) elicited potent anti-leukemic activity in vivo using several AML models, including a patient-derived xenograft (PDX), without evidence of systemic toxicity (Fig. 2C) [99].

Together, these findings identify glycoRNA–csRBP nanoclusters, including those made by csNPM1, as a novel antigenic platform with promising therapeutic potential against a range of hematological and solid malignancies of unmet medical need.

## Conclusion

RNA modifications have recently emerged as central regulators in hematopoietic cell fate and malignant transformation of hematopoiesis. Advances in epitranscriptomic profiling and functional genomics technologies have revealed how these chemical marks modulate transcriptional and translational programs across mRNA, tRNA, rRNA, and non-coding RNAs, shaping self-renewal, lineage restriction, immune escape, and therapy resistance. This review has highlighted key enzymatically active epitranscriptomic regulators, including METTL3, METTL1, ADAR1, and DKC1, and their roles in normal and malignant hematopoiesis, summarised in Table 1.

**Table 1:** Epitranscriptomic regulators in AML: modifications, enzymes, and functional roles.

Modification	Regulator(s)	Type	Substrate(s)	Mechanism	AML/LSC Role
m⁶A	METTL3–METTL14 [14, 16, 22, 23]	Writer complex	mRNA [14]	Co-transcriptional m⁶A deposition; regulates mRNA stability, splicing, translation [7, 14, 16]	Required for AML survival; stabilizes oncogenic mRNAs (e.g., *MYC*, *MYB*, *SON*); METTL3 inhibitor in trials [10, 16, 17, 22]
	METTL16 [27]	Writer	ncRNA; mRNA (e.g., *BCAT1, BCAT2*) [27]	Stabilizes metabolic transcripts [27]	Supports AML maintenance; dispensable in normal hematopoiesis [27]
	FTO [14, 18, 19]	Eraser	mRNA [14]	Removes m⁶A to suppress differentiation programs [14, 18]	Maintains *KMT2A*-rearranged AML; represses ATRA-responsive genes [18]
	ALKBH5 [14, 28, 29]	Eraser	mRNA [14]	Removes m⁶A to stabilize oncogenic mRNAs [28, 29]	Upregulated in AML; promotes LSC survival (e.g., via AXL signaling) [28, 29]
	YTHDC1 [25, 30–32]	Reader	mRNA; caRNA [27, 32, 46, 47]	Regulates nuclear splicing, stabilization, and condensate formation [30–32]	Forms nYACs in AML; essential for LSC maintenance [31, 32]
	YTHDF2 [34, 35]	Reader	mRNA [34]	Mediates cytoplasmic decay of m⁶A-modified transcripts [34, 35]	Preserves HSC quiescence; sustains LSC turnover [34, 35]
	IGF2BP2/IGF2BP3 + YBX1 [41, 42, 45]	Reader complex	_mRNA_ [41, 42, 45]	Stabilizes oncogenic mRNAs (e.g., *BCL2*, *MYC, RCC2*) [41, 42, 45]	Maintains AML cell fitness [41, 42, 45]
	RBFOX2 + RBM15 [46, 47]	Reader + adapter	caRNA [46, 47]	Facilitates PRC2 recruitment and transcriptional silencing [46, 47]	Maintains LSC chromatin state [46, 47]
m⁵C	NSUN2 [52, 60, 61, 67]	Writer	mRNA; tRNA; caRNA [52, 60, 61, 67]	Stabilizes transcripts [52, 60, 61, 67]	Low in *TET2*-mutant and*SRSF2* P95H AML [60, 61]
	TET2 [61, 63, 67, 71]	Eraser	mRNA; caRNA [61, 63, 67, 71]	Oxidizes m⁵C to hm⁵C; reduces mRNA and caRNA stability [61, 63, 67, 71]	Loss stabilizes *TSPAN13*; expands LSCs [71]
	YBX1 [71]	Putative reader	mRNA [71]	Stabilizes transcripts (e.g., *TSPAN13*) [71]	Promotes LSC homing and self-renewal [71]
Ψ	DKC1 [85, 86]	Writer	rRNA; TERC [85, 86]	Catalyzes site-specific pseudouridylation; stabilizes ribosomes and telomerase [85, 86]	Loss impairs translation fidelity and telomerase activity [85, 86]
ac⁴C	NAT10^77^	Writer	mRNA; tRNA; rRNA [77]	Enhances translation via CDS acetylation [78]	Upregulated in AML and MM; enhances translation of *CEP170* and *BCL2L1* (BCL-XL) mRNAs [80, 82–84]
m⁷G	METTL1–WDR4 [69, 70]	Writer complex	tRNA (e.g., tRNA^Arg-TCT^) [71]	Enables AGA codon decoding; increases translation efficiency [71]	Enhances translation of *CDK4* and *HMGA2* mRNAs; associated with adverse prognosis [70, 71]
A-to-I	ADAR1 [94, 95]	Editor	dsRNA [94]	Converts adenosine to inosine (A-to-I) within dsRNA [94]	Induces STAT3β; promotes LSC survival and immune evasion [94, 95]
GlycoRNA	Unknown (non-canonical) [96–98]	Unknown	small ncRNA [96]	Sialylated glycoRNAs displayed at the cell surface; mediate immune recognition [97, 98]	csNPM1 reported on AML blasts; potential immunotherapy target [99]

A summary of RNA modifications implicated in acute myeloid leukemia (AML) and leukemic stem cell (LSC) biology, alongside their associated writers, readers, and erasers, RNA substrates, mechanisms of action, and disease relevance.

*caRNA* chromatin-associated RNA, *CDS* coding sequence, *dsRNA* double-stranded RNA, *ncRNA* non-coding RNA, *LSC* leukemic stem cell, *nYACs* nuclear YTHDC1–m⁶A condensates, *HSC* hematopoietic stem cell, *m⁵C* 5-methylcytosine, *m⁶A* N⁶-methyladenosine, *Ψ* pseudouridine, *m⁷G* N⁷-methylguanosine, *ac⁴C* N⁴-acetylcytidine, *TERC* telomerase RNA component, *ATRA* all-trans retinoic acid, *PRC2* polycomb repressive complex 2, *MM* multiple myeloma, *csNPM1* cell-surface nucleophosmin 1.

Clinical translation is now gaining momentum. The METTL3 inhibitor STC-15 has entered early-phase clinical trials (NCT05584111, NCT06975293) in patients with advanced malignancies, while preclinical efforts targeting METTL1, ADAR1, and DKC1 are also advancing. Early combinatorial strategies, such as METTL3 inhibition alongside immune checkpoint inhibitors, demonstrated therapeutic synergy in preclinical studies [13]. Together, these approaches suggest that epitranscriptomic interventions are poised to complement established AML treatment regimens.

RNA modifications such as m⁶A are under active investigation as functional biomarkers for minimal residual disease (MRD), particularly in chemoresistant AML, where persistent epitranscriptomic alterations may precede clinical relapse. This may be especially valuable in patients lacking robust genetic or immunophenotypic MRD markers [101]. In parallel, targeting cell surface RNA–protein complexes and glycoRNAs offers novel immunotherapeutic opportunities and may facilitate antigen discovery relevant to cellular therapies. Antisense oligonucleotides directed against oncogenic snoRNAs or chromatin-associated RNAs provide an additional layer of selectivity that could further expand the diagnostic and therapeutic toolkit.

Key priorities moving forward include clarifying the context specificity of RNA modifications across disease stages and cellular states, delineating their crosstalk with chromatin and metabolic networks, and embedding epitranscriptomic signatures within existing risk stratification frameworks. Advances in single-cell and locus-specific technologies are expected to map these programs with unprecedented resolution and uncover new therapeutic vulnerabilities. Indeed, the recent discovery of csNPM1 and its glycoRNA–csRBP nanoclusters could potentially help the deeper characterization and better detection of the different normal and malignant hematopoietic cell states, including those identified in the HSPC and LSC fractions. Together, these insights position the epitranscriptome as a mechanistically rich and clinically actionable dimension of hematological malignancies, with broad implications for biomarker development, risk stratification, and targeted intervention.
